# Genome-wide gene expression array identifies novel genes related to disease severity and excessive daytime sleepiness in patients with obstructive sleep apnea

**DOI:** 10.1371/journal.pone.0176575

**Published:** 2017-05-17

**Authors:** Yung-Che Chen, Kuang-Den Chen, Mao-Chang Su, Chien-Hung Chin, Chung-Jen Chen, Chia-Wei Liou, Ting-Wen Chen, Ya-Chun Chang, Kuo-Tung Huang, Chin-Chou Wang, Ting-Ya Wang, Jen-Chieh Chang, Yong-Yong Lin, Yi-Xin Zheng, Meng-Chih Lin, Chang-Chun Hsiao

**Affiliations:** 1 Division of Pulmonary and Critical Care Medicine, Department of Medicine, Kaohsiung Chang Gung Memorial Hospital and Chang Gung University College of Medicine, Kaohsiung, Taiwan; 2 Sleep Center, Kaohsiung Chang Gung Memorial Hospital and Chang Gung University College of Medicine, Kaohsiung, Taiwan; 3 Department of Medicine, Chang Gung University, Taoyuan, Taiwan; 4 Center of Translational Research in Biomedical Sciences, Kaohsiung Chang Gung Memorial Hospital and Chang Gung University College of Medicine, Kaohsiung, Taiwan; 5 Chang Gung University of Science and Technology, Chia-yi, Taiwan; 6 Division of Rheumatology, Kaohsiung Chang Gung Memorial Hospital and Chang Gung University College of Medicine, Kaohsiung, Taiwan; 7 Department of Neurology, Kaohsiung Chang Gung Memorial Hospital and Chang Gung University College of Medicine, Kaohsiung, Taiwan; 8 Molecular Medicine Research Center, and Bioinformatics Center, Chang Gung University, Taoyuan, Taiwan; 9 Graduate Institute of Clinical Medical Sciences, College of Medicine, Chang Gung University, Taoyuan, Taiwan; 10 Center for Shockwave Medicine and Tissue Engineering, Kaohsiung Chang Gung Memorial Hospital, Kaohsiung, Taiwan; Vanderbilt University Medical Center, UNITED STATES

## Abstract

We aimed to identify novel molecular associations between chronic intermittent hypoxia with re-oxygenation and adverse consequences in obstructive sleep apnea (OSA). We analyzed gene expression profiles of peripheral blood mononuclear cells from 48 patients with sleep-disordered breathing stratified into four groups: primary snoring (PS), moderate to severe OSA (MSO), very severe OSA (VSO), and very severe OSA patients on long-term continuous positive airway pressure treatment (VSOC). Comparisons of the microarray gene expression data identified eight genes up-regulated with OSA and down-regulated with CPAP treatment, and five genes down-regulated with OSA and up-regulated with CPAP treatment. Protein expression levels of two genes related to endothelial tight junction (AMOT P130, and PLEKHH3), and three genes related to anti-or pro-apoptosis (BIRC3, ADAR1 P150, and LGALS3) were all increased in the VSO group, while AMOT P130 was further increased, and PLEKHH3, BIRC3, and ADAR1 P150 were all decreased in the VSOC group. Subgroup analyses revealed that AMOT P130 protein expression was increased in OSA patients with excessive daytime sleepiness, BIRC3 protein expression was decreased in OSA patients with hypertension, and LGALS3 protein expression was increased in OSA patients with chronic kidney disease. In vitro short-term intermittent hypoxia with re-oxygenation experiment showed immediate over-expression of ADAR1 P150. In conclusion, we identified a novel association between AMOT/PLEKHH3/BIRC3/ADAR1/LGALS3 over-expressions and high severity index in OSA patients. AMOT and GALIG may constitute an important determinant for the development of hypersomnia and kidney injury, respectively, while BIRC3 may play a protective role in the development of hypertension.

## Background

Obstructive sleep apnea (OSA) has been independently associated with endothelial dysfunction which may explain the increased risk for cardiovascular events, hypertension, and all-cause mortality in this population through chronic intermittent hypoxia with re-oxygenation (IHR) injury[1,2]. A recent meta-analysis has demonstrated that continuous positive airway pressure (CPAP) treatment significantly improved endothelial function as assessed by flow-mediated dilation[3]. It has been observed that circulating leukocytes of OSA patients exhibited markedly enhanced in vitro release of superoxide radical anions and delayed apoptosis, which may in turn lead to endothelial dysfunction[4,5]. However, underlying mechanisms by which IHR leads to endothelial dysfunction and other adverse consequences, such as hypertension, excessive daytime sleepiness (EDS), and chronic kidney disease (CKD), in OSA are largely unknown.

The aim of this study was to explore which expression signatures in peripheral blood could be representative of, or associated with OSA, by investigating the expressions of genes in peripheral blood mononuclear cells (PBMCs) that may be involved in these effects. We hypothesized that the gene expressions of PBMCs involved in the responses to chronic IHR in OSA patients would be markedly different from those in subjects with primary snoring, and that additional differences would be seen between OSA patients with and without hypertension or between OSA patients with and without EDS. Furthermore, we aimed to improve the understanding of the molecular signatures that can correlate endothelial dysfunction with long-term and sufficient CPAP treatment, with the hope that novel genes may be found to be over- or under-expressed after treatment.

A few studies have applied DNA microarray technology to investigate gene expressions in patients with OSA[6–9]. In one of these studies, which focused on gene expressions in the blood leukocytes from adult OSA patients, genes involved in modulation of reactive oxygen species, cell growth, proliferation, and the cell cycle were found to be altered after one night of sleep in four OSA patients free of any co-morbidity[6]. The extent to which the leukocyte genes play a role in OSA patients with adverse consequences, and the effect of nasal CPAP on gene signatures is unclear. Therefore, we extended our investigation into OSA patients with long-term CPAP treatment, hypertension, or EDS by analyzing whole-genome gene expression profiles of PBMC in three comparisons: (1) treatment-naïve moderate to very severe OSA patients versus subjects with primary snoring; (2) moderate to very severe OSA patients with hypertension or EDS versus those without hypertension or EDS, respectively; (3) treatment-naïve very severe OSA patients versus those receiving at least one year of adequate CPAP treatment. We identified a novel association between several genes related to apoptosis or endothelial function and OSA or its clinical phenotypes.

## Materials and methods

### Subjects

The study was approved by the Institutional Review Board of Chung Gung Memorial Hospital, Taiwan. The study participants were recruited from the pulmonary clinics and health examination center of Kaohsiung Chung Gung Memorial Hospital January 2012 through December 2014. Written informed consent was obtained from each subject participating in the study, who was aged 20 years or older. All study participants underwent full-night in-laboratory polysomnography examinations as described previously, and OSA was diagnosed according to the AASM guideline[10]. The exclusion criteria included ongoing infections, autoimmune disease, use of immunosuppressive agent in the past 6 months, narcolepsy, severe obesity (body mass index, BMI, ≧35 kg/m^2^), old age (>65 year-old), and those with a BMI < 21 kg/m^2^. Study cohorts used for the whole-genome microarray gene expression and protein expression experiments included 48 (cohort 1) and 68 subjects (cohort 2: cohort 1 with expanded sample size), respectively. All these participants were classified into the following four groups based on apnea hypopnea index (AHI) and long-term use of CPAP: subjects with primary snoring (PS; AHI<5), treatment-naïve patients with moderate to severe OSA (MSO; 15<AHI≦50), treatment-naïve patients with very severe OSA (VSO; AHI>50), and very severe OSA patients on long-term CPAP treatment (VSOC; AHI>50 and regular use of CPAP: >4 hours/night, > one year). Nocturnal hypoxemia was evaluated in terms of the percentage of total minutes of recording time with oxyhemoglobin saturation <90% (%time <90% SaO2), and the number of dips >4% of basal SaO2%//h (oxygen desaturation index, ODI). The Epworth Sleepiness Scale (ESS) recorded at the PSG exam was used to measure sleep propensity, and EDS was defined as ESS>10. Hypertension was defined as baseline blood pressure>140/90 mmHg. Heart disease included ischemic heart disease, cardiac arrythmia, and congestive heart failure. CKD was defined as estimated glomerular filtration rate <60 mL/min/1.73 m^2^ for ≥ 3 months.

### Processes of RNA isolation and cRNA synthesis

Peripheral whole blood (20 ml) was collected at AM 6:00 to 8:00 after written informed consent was obtained from all the recruited participants. The PBMCs were isolated by Ficoll-Hypaque gradient centrifugation (HISTOPAQUE^®^-119, Sigma-Aldrich, Inc., St. Louis, MO USA) within 90 min of drawing blood, washed in PBS, and then stored in RNAlater (Ambion Inc., Austin, TX, USA) at -80°C until RNA isolation. An RNeasy^®^ Plus Mini Kit (Qiagen, Hilden, Germany) was used for isolation of high quality total RNA, and treated with DNase according to the manufacture protocol. RNA samples were run on a RNA 6000 Nano Gel System (Agilent Technologies Inc., Palo Alto, CA, USA) using an Agilent 2100 Bioanalyzer (Agilent) to determine the quality of RNA. Only samples with A260/A280 ratios of 1.9 to 2.1 were used for further analysis. A total of 300 ng RNA was used for synthesis of first strand cDNA and transcription of cRNA using an Illumina Totalprep RNA Amplication kit (Ambion, Inc.).

### Gene expression profiling and microarray data analysis in the study cohort 1

Illumina (San Diego, CA) HumanRef-12-version 2 bead microarrays were used with 750 ng labeled cRNA for each sample according to the manufacturer's protocol. Human Ref-12 version 2 arrays consist of 27,455 probes representing 21,910 unique human genes on an eight-strip format array. All expression dataset has been deposited in the NCBI Gene Expression Omnibus with accession number of GSE75097.The preprocessing, quality control, background subtraction, quantile normalization and log2 transformation of array data for cross comparison were performed using BeadStudio software. Statistical analysis of the microarray data was further performed, using GeneSpring software version 11 (Agilent Technologies Inc., Santa Clara, CA, USA) as previously described[11]. Because the sample sizes were relatively small and statistics could be influenced by outliers, a non-parametric U test for unpaired comparisons of the two independent groups was applied. The Benjamini-Hochberg false-discovery rate correction method was used for controlling false positives and a corrected p-value cutoff of 0.05 was used to select the sets of significantly up- and down-regulated genes. The gene sets were then grouped into functional categories according to the Gene Ontology Biological Processes Classification. The pathway enrichment process was used to find direct relationships between genes of interest. This was performed in GeneSpring software with the “simple and direct interaction” algorithm.

### Measurement of protein expression levels of five selected genes from PBMC samples in the study cohort 2

PBMCs were lysed in radio-immuno-precipitation assay-buffer containing a protease inhibitor cocktail (Sigma-Aldrich). Protein lysate normalized to 20 ng total protein was used to measure angiomotin (AMOT) P130, pleckstrin homology domain containing, family H member 3 (PLEKHH3), baculoviral IAP repeat-containing 3 (BIRC3; LGALS3), adenosine deaminase RNA-specific (ADAR1) P150, and galectin-3 internal gene (GALIG) levels by a commercial enzyme-linked immunosorbent assay (ELISA) kit, where AMOT kit was from USCN Business Co. (China), PLEKHH3 kit was from Sunlong (China), BIRC3/ADAR kits were from Cusabio (China), and LGALS3 kit was from R&D Systems (Minneapolis, MN). Briefly, 40 μl dye (Bio-Rad Protein Assay Dye Reagent Concentrate #500–0006) was added to 10 μl bovine serum albumin with serial dilution to generate a standard curve for total protein concentration by measuring values at OD 595nm.

### In vitro blood cell culture under IHR conditions

PMBC from six healthy subjects (600 μL per well, and adjusted to 1×10^6^ cells per ml) were exposed to normoxia (NOX) or IHR in a custom-designed, incubation chambers which are attached to an external O2-CO2-N2 computer-driven controller, as described previously[12]. Air-phase set point consisted of a 35-min hypoxic period (0% O2 and 5% CO2), followed by 25 min of re-oxygenation (21% O2 and 5% CO2), using the BioSpherix OxyCycler C42 system (BioSpherix, Redfield, NY), 7 hours each day for 4 days. Control cells were maintained in NOX conditions for the same durations. Previous studies have shown that a 30–40% decreases in blood SaO2 could be achieved in the conditioned media by 25 min of continuous exposure of cells to 0%O2 and 5%CO2[13,14]. Protein expression levels of the five selected genes in the PBMC samples were determined using ELISA method, as described above.

### Statistical analysis

Continuous values were presented as the mean ± standard deviation (SD). ANOVA test followed by post hoc analysis with Bonferroni test was used for comparing mean values of more than two experimental groups in case of homogeneous data, while Brown-Forsythe test followed by post hoc analysis with Games-Howell test was used in case of non-homogeneous data. Chi-square tests were used to assess the differences of category values between different groups. In subgroup analyses, multivariate linear regression model was used to adjust for confounding factors, including age, gender, BMI, co-morbidities (hypertension, diabetes mellitus, stroke, heart disease, and CKD), smoking and alcoholism history, and to obtain adjusted p values. Pearson correlation test was used to assess the correlation between two continuous variables. All tests were two tailed and the null hypothesis was rejected at p < 0.05. A statistical software package (SPSS, version 15.0, Chicago, IL) was used for all analyses.

## Results

### Demographic data of the participants

The baseline, sleep, and biochemistry data of the study cohort 1 and 2 are listed in **Table 1**. The study population was all residents in Taiwan. Age, BMI, male gender ratio, smoking history, alcoholism history, and co-morbidities were all matched among the four subgroups, except that more patients in the VSOC group had diabetes mellitus than those in the other 3 groups in the study cohort 2.

**Table 1:** Demographic, sleep, and laboratory data of study participants in the microarray gene expression experiment (study cohort 1 and 2).

	Study Cohort 1					Study Cohort 2				
	Subjects with primary snoring (PS) N = 6	Moderate-severe OSA patients (MSO) N = 15	Very severe OSA patients(VSO) N = 12	Very severe OSA patients on CPAP (VSOC) N = 15	P value	Subjects with primary snoring(PS) N = 16	Moderate-severe OSA patients (MSO) N = 18	Very severe OSA patients (VSO) N = 18	Very severe OSA patients on CPAP (VSOC) N = 16	P value
Age, years	51.0±11.3	46.9±11.9	47±11.4	50.4±8	0.592	49.7±9.3	47.6±11.5	47.5±10.2	50.4±8.0	0.766
Male Sex, n(%)	4 (66.7)	12 (80)	9 (81.8)	12 (75)	0.871	8 (50)	14 (77.8)	16 (88.9)	12 (75)	0.075
BMI, kg/m^2^	25.1±2	27.2±3.8	28±3.6	28.4±4.5	0.231	26.6±3.4	27.2±3.8	26.9±4.5	28.3±4.5	0.647
AHI,events/hour	4.2±2.1	34.1±8.8	67.4±12.3	66.2±24.3	<0.001	3.1±2.5	34.8±9.5	70.3±12.1	64.5±24.5	<0.001
ODI,events/hour	2.4±1.4	24.9±13.8	62.1±11.9	58.1±26.4	<0.001	2.1±2.0	23.9±13.2	59.1±22.1	58.1±26.4	<0.001
Mean SaO2, %	96.8±0.4	95.2±1.9	93.3±2.2	93.2±3.6	<0.001	97.2±0.7	95.3±1.8	93.6±2.3	93.2±3.6	<0.001
Minimum SaO2, %	88.5±1.8	78.3±8.5	65.5±12.7	72±11.3	<0.001	89.3±3.4	78.9±8.2	67.6±12.9	72.0±11.3	<0.001
Arousal index	10.9±9.6	30.5±24.4	46.6±29.5	63.6±41.9	0.002	13.9±10.5	32.9±25.1	46.9±27.3	63.6±41.9	<0.001
ESS	12.3±5.9	8.7±5.4	10.5±4.7	14.1±4.7	0.026	6.8±6.2	8.4±5.4	11.1±5.1	14.1±5.7	0.003
Smoking, n(%)	5 (83.3)	9 (60)	6 (54.5)	11 (68.8)	0.612	2 (14.3)	7 (38.9)	7 (38.9)	5 (31.3)	0.421
Alcoholism, n(%)	5 (83.3)	7 (46.7)	8 (72.7)	11 (68.8)	0.322	0 (0)	2 (11.1)	2 (11.1)	0 (0)	0.315
Cholesterol, mg/dl	190.2±36.2	203.1±47.3	174.6±59.3	164.4±71.5	0.356	190.2±39.6	187.6±66.4	184.6±53.8	164.5±71.5	0.621
Triglycerides, mg/dl	130.8±67	199±126.4	155.5±59.9	194.6±151.9	0.499	120.6±56.8	194.8±121.1	155.4±60.1	194.6±151.9	0.153
HDL, mg/dl	59.8±14.5	52.7±10.9	49.9±4.7	48.8±18.4	0.193	57.5±11.2	49.1±16.1	52.7±9.5	48.7±13.4	0.321
LDL, mg/dl	106.7±33.6	110.1±31.6	102.5±31.7	100.4±40.1	0.956	102.2±31.4	110.7±33.1	107.2±38.1	100.4±40.1	0.866
Glucose, mg/dl	96.3±5.8	101.3±15.7	101.6±12.3	107.9±25.5	0.355	94.8±5.7	99.7±15.7	97.2±13.7	107.9±25.4	0.194
Co-morbidity,n(%)										
Hypertension	2 (33.3)	6 (40)	2 (18.2)	8 (50)	0.271	4 (25)	7 (38.9)	6 (33.3)	8 (50)	0.516
Diabetes mellitus	0 (0)	0 (0)	0 (0)	3 (18.8)	0.071	0 (0)	0 (0)	0 (0)	3 (18.8)	0.017
Cardiac disease	1 (16.7)	1 (6.7)	1 (9.1)	2 (12.5)	0.752	2 (12.5)	2 (11.1)	2 (11.1)	2 (12.5)	0.999
Stroke	1 (16.7)	0 (0)	0 (0)	0 (0)	0.067	1 (6.3)	0 (0)	0 (0)	0 (0)	0.348
Chronic kidney disease	1 (16.7)	1 (6.7)	1 (8.3)	2 (13.3)	0.384	1 (6.3)	1 (5.6)	2 (11.1)	2 (12.5)	0.86

BMI = body mass index; AHI = apnea hypopnea index; ODI = oxygen desaturation index; HDL = high density lipoprotein; LDL = low density lipoprotein

#### Differentially expressed genes (DEG) identified in the whole-genome gene expression experiment in the validation cohort 1

**Fig 1:**
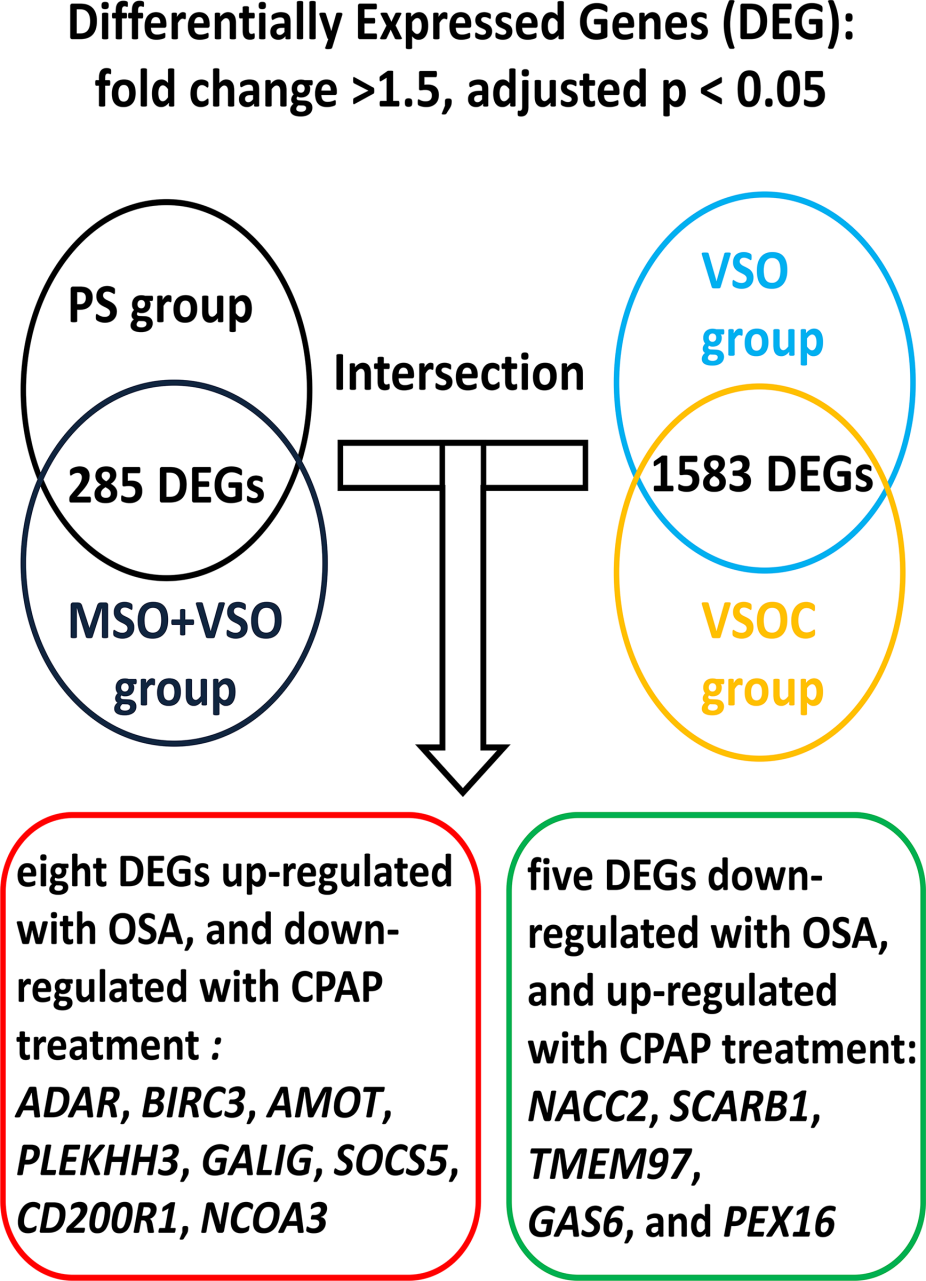
Flow chart for identifying candidate differentially expressed genes through the intersection of comparison I and II. Differentially expressed genes were identified from the intersection of the comparisons between treatment-naïve OSA versus PS (comparison I) and treatment-naïve OSA versus OSA patients with long-term CPAP treatment (comparison II).Microarray identified 13 differentially expressed genes (DEG) regulated in the opposite direction for the two comparisons.

**Fig 2:**
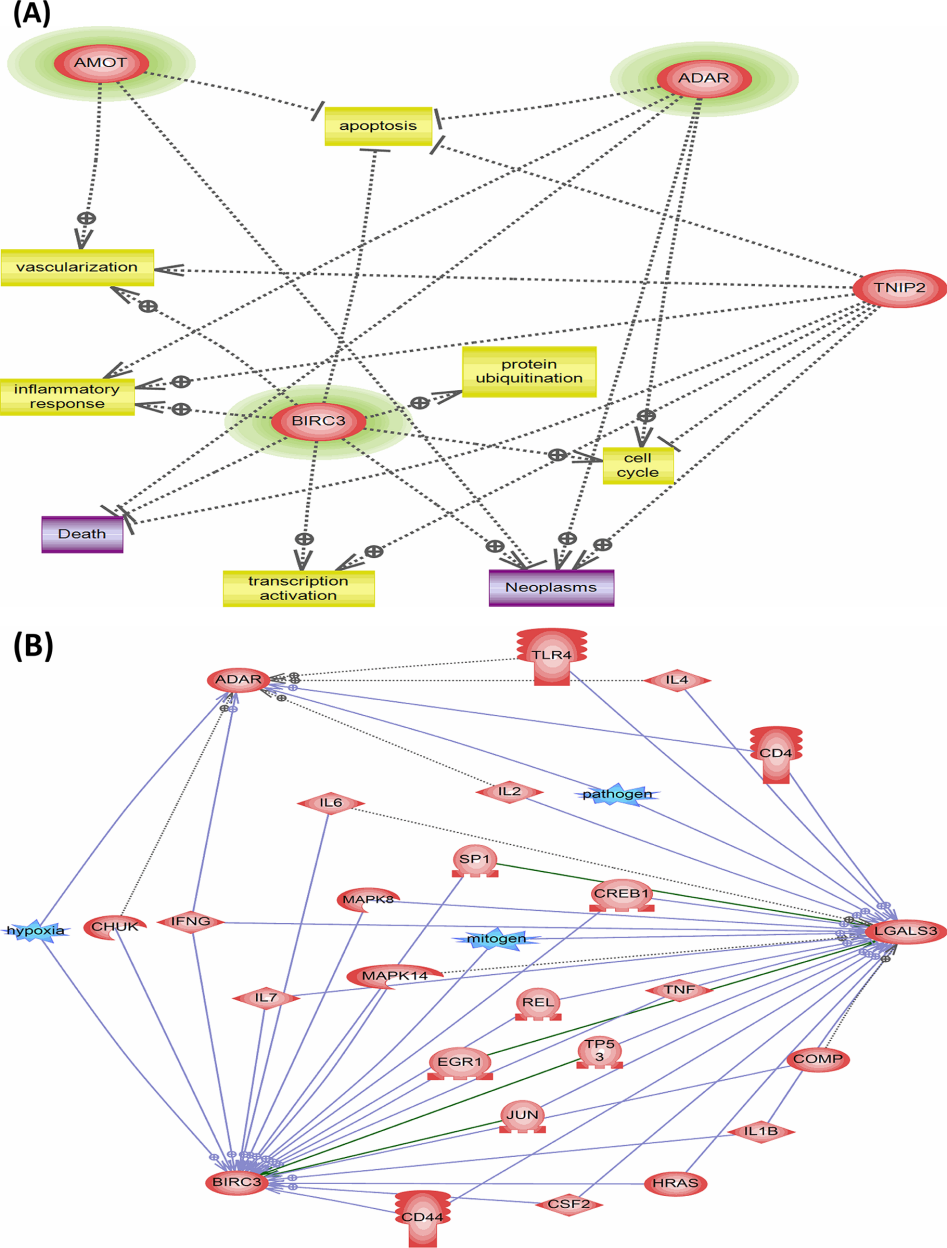
OSA-regulated signaling pathways and gene interactions based on the analyses of the microarray gene expression data. (A) OSA-regulated signaling pathways were involved in apoptosis, angiogenesis, and tight junctions. Differentially expressed genes up-regulated with OSA and down-regulated with CPAP treatment are shown in green colored circles. (B) Gene interactions among the candidate differentially expressed genes.

Microarrays from the 48 subjects that passed the quality-control filters were included in this study. From the 22,150 probes, 285 transcripts were differentially expressed between the treatment-naïve OSA patients (MSO and VSO groups) and PS group using a Mann-Whitney unpaired test (comparison I). To assess the effect of long-term CPAP treatment on PBMC, gene expression profiles in the VSOC group was compared with that in the VSO group, and differential expression of 1583transcripts was identified (comparison II). The intersection of comparison I and II result in eight DEG up-regulated with OSA and down-regulated with CPAP treatment, and five DEG down-regulated with OSA and up-regulated with CPAP treatment (**Fig 1** and **Table 2**). Gene Ontology and gene interaction analyses revealed that *AMOT*, *BIRC3*, and *ADAR* are all involved in anti-apoptosis signaling, and *LGALS3* serves as pro-apoptosis molecule, while *AMOT*, *BIRC3*, and *PLEKHH3* are all involved in angiogenesis or tight junction (**Fig 2A and 2B**). To further clarify the effects of hypertension or EDS on the gene signature of PBMCs, gene expression profiles in the treatment-naïve OSA patients with hypertension or EDS was compared with those without hypertension or EDS, respectively. Six genes, including *AMOT* and *PLEKHH3*, were up-regulated (**Table 3**) in the treatment-naïve OSA patients versus PS subjects and further up-regulated in OSA patients with EDS as compared to that in those without EDS.*LGALS3*(fold change 2.23, p = 0.003) and *metallopeptidase inhibitor 2* (*TIMP2*; fold change 4.44, p = 0.043) were up-regulated in the treatment-naïve OSA patients versus PS subjects, and further up-regulated in OSA patients with hypertension(LGALS3: fold change 4.37, p = 0.044; *TIMP2*; fold change 5.42, p = 0.018) as compared to that in those without hypertension (**Table 3**).

**Table 2:** Differentially expressed genes associated with OSA and reversed with CPAP treatment in the microarray gene expression experiment.

Gene Name	Probe ID	Treatment-naïve OSAversus PS subjects		VSOC versus VSO patients		Description
		Fold Change	P value	Fold change	P value	
*ADAR*	5130612	6.8036	0.00561	0.29877	0.04467	Adenosine deaminase, RNA-specific variant 4.
*BIRC3*	3060255	1.5465	0.02985	0.71495	0.04965	Baculoviral IAP repeat-containing 3, variant 2.
*AMOT*	3290646	2.00353	0.01754	0.77545	0.03214	Angiomotin, variant 2.
*PLEKHH3*	1070709	4.23447	0.03055	0.31054	0.04834	Pleckstrin homology domain containing, family H member 3.
*LGALS3*	1660647	3.87876	0.02832	0.29394	0.0462	Galectin-3 internal gene.
*SOCS5*	4880750	1.51511	0.03722	0.15028	0.00193	Suppressor of cytokine signaling 5, variant 2.
*CD200R1*	4280523	2.10105	0.02045	0.35276	0.00151	CD200 receptor 1, variant 3.
*NCOA3*	2760390	1.31953	0.01321	0.73542	0.0086	Nuclear receptor coactivator 3, variant 1.
*NACC2*	4220739	0.36967	0.0464	2.5467	0.00732	NACC family member 2, BEN and BTB (POZ) domain containing.
*SCARB1*	1170338	0.59278	0.01744	1.6449	0.02541	Scavenger receptor class B, member 1.
*TMEM97*	3890561	0.70155	0.02856	1.31361	0.01644	Transmembrane protein 97.
*GAS6*	70730	0.53071	0.0155	1.63026	0.0131	Growth arrest-specific 6.
*PEX16*	2750358	0.57187	0.01781	1.38059	0.01495	Peroxisomal biogenesis factor 16, variant 2.

OSA = obstructive sleep apnea; CPAP = continuous positive airway pressure; VSO = very severe OSA patients; VSOC = very severe OSA patients on long-term CPAP treatment; PS = primary snoring subjects

**Table 3:** Differentially expressed genes up-regulated with obstructive sleep apnea (OSA) and further up-regulated with excessive daytime sleepiness (EDS) or hypertension (HT).

Gene Name	Probe ID	Treatment-naïve OSA versus primary snoring subjects		OSA patients with EDS or HT versus those without EDS or HT		Description
		Fold Change	P value	Fold change	P value	
Genes up-regulated with both OSA and EDS						
*AMOT*	3290646	2.00353	0.01754	1.66391	0.03214	Angiomotin, transcript variant 2.
*LAMB3*	730040	4.29633	0.02958	3.40345	0.0451	Laminin, beta 3, transcript variant 1.
*SEC14L2*	4890341	3.76125	0.02942	3.73724	0.04485	SEC14-like 2 (S. cerevisiae).
*ITFG3*	6250553	1.34628	0.03237	1.29703	0.04873	Integrin alpha FG-GAP repeat containing 3.
*HIF1A*	2680722	1.31563	0.04622	2.62267	0.04917	Hypoxia-inducible factor 1, alpha subunit (basic helix-loop-helix transcription factor), transcript variant 2.
*PLEKHH3*	107079	4.23447	0.03055	2.75882	0.02976	Pleckstrin homology domain containing, family H (with MyTH4 domain) member 3.
Genes up-regulated with both OSA and HT						
*LGALS3*	1660647	2.23	0.03	4.37	0.044	Galectin-3 internal gene.
*TIMP2*	5420743	4.44	0.043	5.42	0.018	metallopeptidase inhibitor 2

### Differential protein expression levels of the five selected genes in the study cohort 2

**Fig 3:**
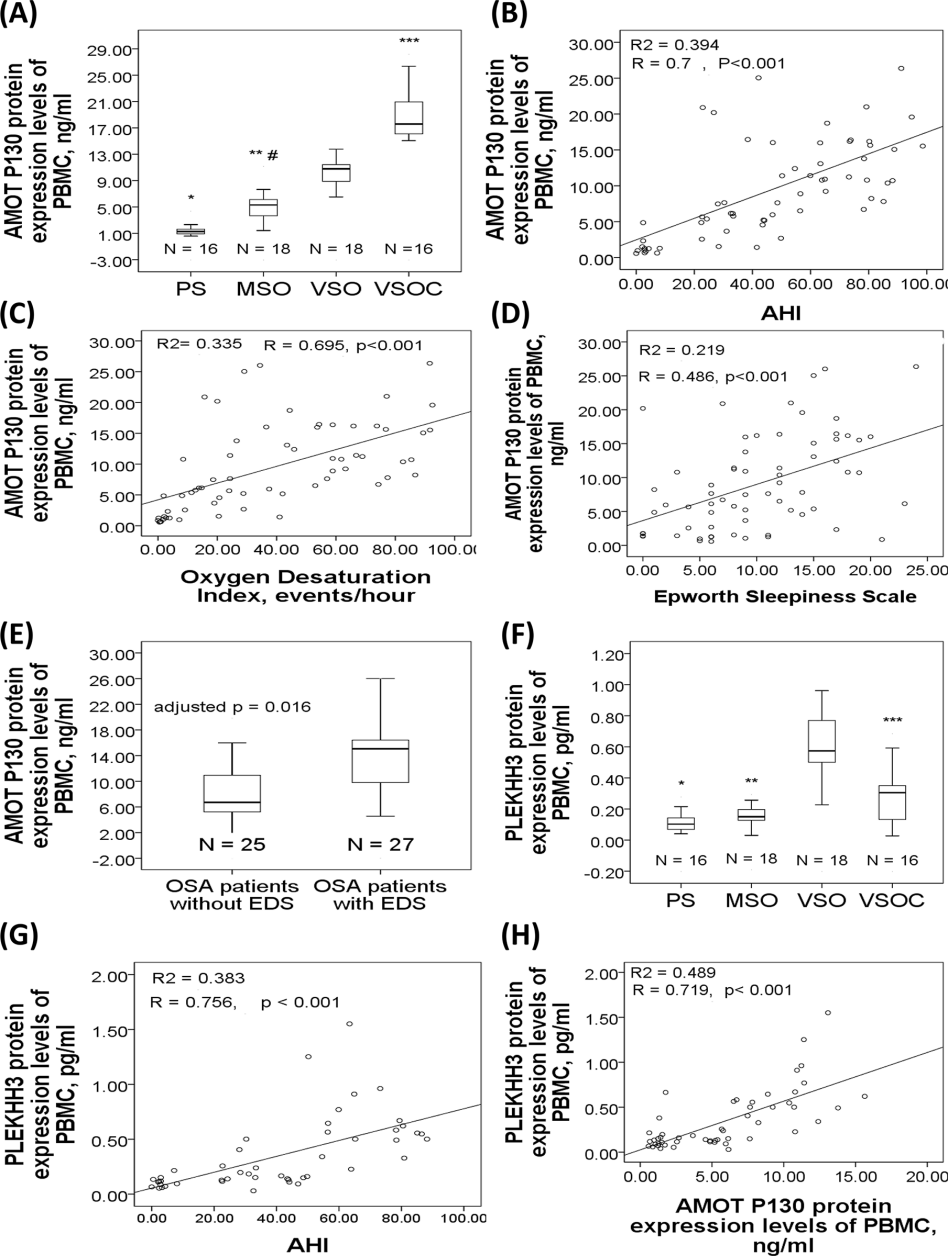
AMOT P130 and PLKHH3 protein expression levels in the study cohort 2. (A) AMOT P130 protein expression was increased in treatment-naïve moderate to severe (MSO) and very severe OSA (VSO) patients as compared to that in primary snoring (PS) subjects, and further increased in very severe OSA patients with long-term CPAP treatment (VSOC). AMOT P130 protein expression was positive correlated with (B) apnea hypopnea index, (C) oxygen desaturation index, and (D) Epworth Sleepiness Scale. (E) AMOT P130 protein expression was increased in OSA patients with excessive daytime sleepiness. (F) PLEKHH3 protein expression was increased in the VSO group and decreased in the VSOC group. PLEKHH3 protein expression was positively correlated with (G) AHI and (H) AMOT P130 protein expression. *p<0.001 compared between the VSO and PS groups. **P<0.001, compared between the VSO and MSO groups. ***P<0.001, compared between the VSO and VSOC groups. #p<0.001, compared between the MSO and PS groups.

Analysis of the variance revealed significant between-group differences in AMOT (p<0.001), PLEKHH3 (p<0.001), BIRC3 (p = 0.001), ADAR1 (p<0.001), and LGALS3 (p = 0.017) protein expression levels. Post hoc analyses with corrections for multiple comparisons revealed that patients in both the MSO (4.97±1.92 ng/ml, p<0.001) and VSO (10.55±29.72.38 ng/ml, p<0.001) groups had significantly increased AMOT P130 protein expression than the PS group (1.46±1.01 ng/ml), and that patients in the VSO group had higher AMOT P130 protein expression than those in the MSO group (p<0.001), while patients in the VSOC group (19.1±3.86 ng/ml, p<0.001) had even higher AMOT P130 protein expression than those in the VSO group (**Fig 3A**).AMOT P130 protein expression was positively correlated with AHI (r = 0.7, p<0.001, **Fig 3B**), ODI (r = 0.695, p<0.001, **Fig 3C**), percentage time with SaO2<90% (r = 0.585, p<0.001), and ESS (r = 0.476, p<0.001, **Fig 3D**). Subgroup analysis showed that OSA patients with EDS had significantly increased AMOT P130 protein expression than those without EDS (14.05±6.19 versus 8.23±5.22 ng/ml, adjusted p = 0.016, **Fig 3E**). PLEKHH3 protein expression in the VSO group (0.67±0.33 pg/ml) was significantly increased as compared to that either in the PS (0.15±0.16 pg/ml, p<0.001), MSO (0.18±0.11 pg/ml, p<0.001), or VSOC (0.29±0.17 pg/ml, p<0.001) group (**Fig 3F**).PLEKHH3 protein expression level was positively correlated with AHI (r = 0.737, p<0.001, **Fig 3G**), ODI (r = 0.681, p<0.001), percentage time with SaO2<90% (r = 0.631, p<0.001), and AMOT P130 protein expression (r = 0.719, p<0.001, **Fig 3H**) in all the treatment-naïve subjects (PS, MSO, and VSO groups).

**Fig 4:**
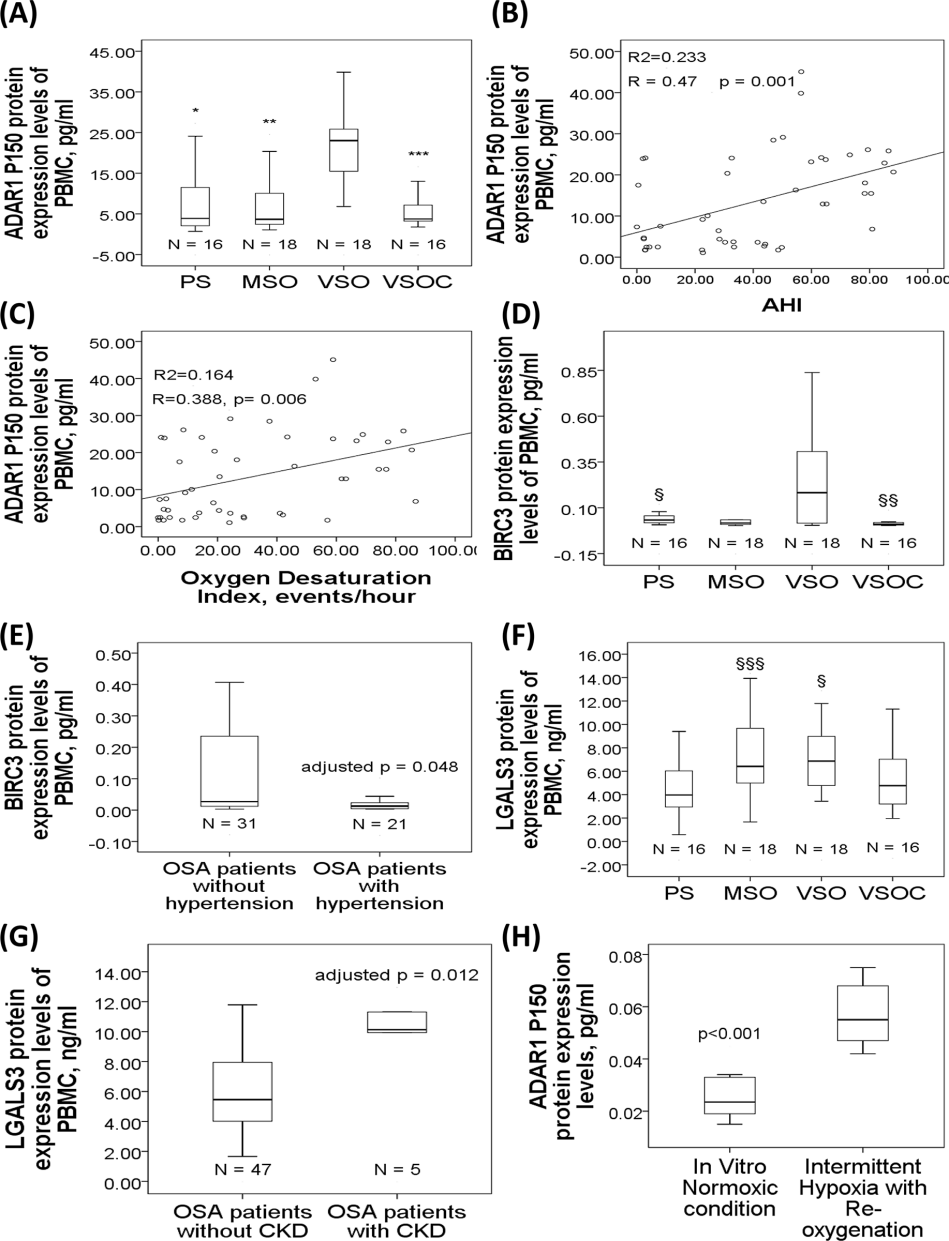
ADAR1 P150, BIRC3, and LGALS3 protein expression levels in the study cohort 2. (A) ADAR1 P150 protein expression was increased in the VSO group, and decreased in the VSCO group. ADAR1 P150 protein expression was positively correlated with (B) AHI and (C) ODI. (D) BIRC3 protein expression was increased in the VSO group and decreased in the VSOC group. (E) BIRC3 protein expression was decreased in OSA patients with hypertension as compared to that in those without hypertension. (F) LGALS3 protein expression was increased in the MSO and VSO groups as compared to that in the PS group. (G) LGALS3 protein expression was increased in OSA patients with chronic kidney disease. (H) ADAR1 P150 protein expression was increased under short-term in vitro intermittent hypoxia with re-oxygenation condition. §p<0.05, compared between the VSO and PS groups. §§p<0.05, compared between the VSO and VSOC groups. §§§p<0.05, compared between the MSO and PS groups.

ADAR1 P150 protein expression level was significantly increased in the VSO group (22.42±9.33 pg/ml) as compared to that either in the PS (7.6±8.03 pg/ml, p<0.001), MSO (7.92±8.33 pg/ml, p<0.001), or VSOC (6.29±6.83 pg/ml, p<0.001) group (**Fig 4A**). ADAR1 P150 protein expression level was positively correlated with AHI (r = 0.47, p = 0.001, **Fig 4B**) and ODI(r = 0.388, p = 0.006, **Fig 4C**). BIRC3 protein expression was significantly increased in the VSO group (0.31±0.39 pg/ml) as compared to that in the PS (0.05±0.05 pg/ml, p = 0.031) or VSOC (0.03±0.05 pg/ml, p = 0.033) group (**Fig 4D**). Subgroup analysis showed that OSA patients with hypertension had significantly deceased BIRC3 protein expression levels as compared to those without hypertension (0.04±0.06 versus 0.21±0.33 pg/ml, adjusted p = 0.048, **Fig 4E**). LGALS3 protein expression level was significantly increased in both the MSO (7.04±3.18 ng/ml, p = 0.036) and VSO groups (4.52±1.07 ng/ml, p = 0.035) as compared to that in the PS group (4.24±2.35 ng/ml) (**Fig 4F**). Subgroup analysis showed that OSA patients with CKD had significantly increased LGALS3 protein expression levels than those without CKD (10.67±3.51 versus 6.4±3.44 ng/ml, adjusted p = 0.012, **Fig 4G**).

### Effects of in vitro IHR on protein expressions of the five selected genes

To determine whether IHR per se can affect expressions of the five selected genes, PBMC from six healthy subjects were exposed in vitro to either 7 cycles of IHR per day for 4 days or 4 days of continuous normoxic condition. Short-term IHR treatment in vitro resulted in a significant increase of ADAR1 protein expression (0.057±0.013 versus 0.025±0.008, p value<0.001, **Fig 4H**) as compared with normoxic condition, whereas no significant change was found for the other 4 protein expressions.

## Discussion

In this study, we identified a novel association between several genes related to apoptosis or endothelial function and OSA or its clinical phenotypes through whole-genome gene expression microarray analyses. Moreover, we verified AMOT, PLEKHH3, ADAR, BIRC3, and LGALS3 protein over-expressions in the treatment-naïve OSA patients, and discovered a correlation between AMOT/BIRC3/LGALS3 protein expression and the presence of EDS/hypertension/CKD, respectively. Furthermore, we found an immediate effect of in vitro IHR on ADAR1 P150 protein over-expression in a cell culture model.

Endothelial tight junctions form a seal between polar cells, isolating the lumen of the blood vessel from the surrounding tissue and restricting the diffusion of solutes from the blood to the surrounding cells. AMOT is a trans-membrane receptor for the angiostatic factor angiostatin. Alternative splicing of AMOT mRNA results in two protein isoforms (p80 and p130), which exert very distinct roles during angiogenesis[15]. AMOT p80 stimulates endothelial cell migration and angiogenesis in response to angiostatin, whereas AMOT p130 localizes to actin and tight junctions[16,17]. AMOT is also involved in the maturation of epithelial tight junctions as part of the zonular signalosome formation[17,18]. In this study, *AMOT* gene and P130 protein expressions were both up-regulated in the treatment-naïve OSA patients, whereas *AMOT* gene expression was down-regulated and P130 protein expression was further up-regulated in the OSA patients with long-term CPAP treatment. We speculate that post-transcriptional modifications, maturation, alternative splicing, and degradation processes, may contribute to this discrepancy. The further increase of AMOT P130 protein expression in the VSOC group may be attributed to long-term air flow stimulation of upper airway epithelial cells with CPAP use. In line with our findings, AMOT expression has been shown to be a good indicator of plasticity of the vascular network in skeletal muscle, and P130 expression can be reduced with exercise training in an obese rat model[19].

Pleckstrin homology (PH) domains family function as a versatile protein–protein interaction platform and are integrated in an increasing number of available multidomain structures[20]. For example, PLEKHA7 is a recently identified protein of the epithelial zonula adhaerens, and stabilizes it by modulating the dynamics of assembly and disassembly of the tight junction barrier, through E-cadherin protein complex- and microtubule-dependent mechanisms[21]. Zonula adhaerens are topologically associated with tight junctions in the apical junctional complex at the apicolateral border of both epithelial and endothelial polar cells. In this study, we found a positive correlation between AHI and PLEKHH3 expression level, which was reversed with CPAP treatment. We think that PLEKHH3, another PH family member, may play a pivotal role in chronic IHR-related endothelial dysfunction in OSA patients through regulating tight junction formation of endothelial cells specifically.

Anti-apoptotic marker, BIRC3, has been shown to mediate the pro-survival and inflammatory responses induced by the docosahexaenoic acid / neuroprotectin D1 pathway under oxidative stress in an ischemia-reperfusion stroke model[22].BIRC3 has been demonstrated to be a downstream effector of HIF-1 signaling involved in the survival response of endothelial cells to hypoxia[23]. BIRC3 is also a negative regulator of the non-canonical NF-κB signaling pathway and mutated primarily in patients with aggressive chronic lymphocytic leukemia[24]. In this study, BIRC3 was up-regulated in the treatment-naive OSA patients, and down-regulated with CPAP treatment. BIRC3 under-expression was associated with the occurrence of hypertension in OSA patients. We speculate that BIRC3 may play a role in protecting from hypertension in OSA patients through inhibiting endothelial cell apoptosis and NF-κB signaling.

Adenosine deaminase acting on RNA1 (ADAR1) catalyzes the C6 deamination of adenosine (A) to produce inosine (I) in regions of double-stranded RNA, known as A-to-I RNA editing. Alternative splicing gives rise to transcripts that encode twoADAR1 protein size isoforms. ADAR1 p150 is an interferon-inducible dsRNA adenosine deaminase found in the cytoplasm and nucleus, mounting pro-viral and anti-apoptotic responses, whereas ADAR1 p110 is constitutively expressed in the nucleus[25]. Among the biologically relevant substrates of ADAR1 that result in amino acid coding changes following editing are transcripts for the 5-HT2c-R neurotransmitter receptor for serotonin[26]. In recent years, this modification has been discovered to occur not only in coding RNAs but also in non-coding RNAs, such as microRNAs, small interfering RNAs, transfer RNAs, and long non-coding RNAs[27].The malfunction of this editing machinery is associated with various human diseases, such as neurodegenerative, cardiovascular, and carcinogenic diseases. For the first time, we found an association between ADAR1 up-regulation and AHI, and demonstrated a direct link between IHR and ADAR1 P150 protein over-expression in vitro.

LGALS3 (GALIG) is a novel cell death gene encoding mitogaligin, a protein promoting cytochrome c release upon direct interaction with the mitochondria or nucleus[28,29].LGALS3 pro-apoptotic gene is up-regulated during neutrophils apoptosis and under-expressed in acute myeloid leukemia cells[30]. In this study, we found that LGALS3 was up regulated in moderate to very severe OSA patients, especially in those with CKD. Impaired renal function has been observed in OSA patients with metabolic syndrome, hypertension or heart failure, possibly through reduced endothelial nitricoxide synthase expression[31,32]. Our findings suggest that LGALS3 may play a crucial role in mediating renal dysfunction in OSA patients.

There are several limitations to the present study. First, the cause and effect relationship could not be determined in this cross-sectional clinical study design, and further studies are required to elucidate underlying mechanisms for these five novel biomarkers. However, the in vitro experiment demonstrated immediate over-expression of ADAR1 P150 under short-term IHR stimuli. Second, gene expression levels of the five selected genes were not examined in the PBMC samples because of inadequate RNA samples. However, their protein expressions showed corresponding changes, indicating a functional role of these novel genes in mediating pathogenesis of OSA and its adverse consequences. Third, the sample size of each subgroup is relatively small, and many confounding factors may affect the expression levels. Further studies with sufficiently large sample sizes are required for the internal and external validity and the reliability of the results.

In summary, we reported a novel association of increased AHI in OSA patients of Asian origin with over-expressions of the *AMOT*, *PLEKHH3*, *ADAR1*, *BIRC3*, and *LGALS3* genes in blood immune cells. The findings extend reports linking AMOT with EDS in OSA patients, and provide direct evidence that perturbation of BIRC3 and LGALS3 signaling may play an important role in the mediation of hypertension and CKD in OSA patients, respectively.
